# Hippo signalling during development

**DOI:** 10.1242/dev.167106

**Published:** 2019-09-16

**Authors:** John Robert Davis, Nicolas Tapon

**Affiliations:** Apoptosis and Proliferation Control Laboratory, The Francis Crick Institute, 1 Midland Road, London NW1 1AT, UK

**Keywords:** Cell fate, Differentiation, Growth, Hippo pathway, Morphogenesis, YAP

## Abstract

The Hippo signalling pathway and its transcriptional co-activator targets Yorkie/YAP/TAZ first came to attention because of their role in tissue growth control. Over the past 15 years, it has become clear that, like other developmental pathways (e.g. the Wnt, Hedgehog and TGFβ pathways), Hippo signalling is a ‘jack of all trades’ that is reiteratively used to mediate a range of cellular decision-making processes from proliferation, death and morphogenesis to cell fate determination. Here, and in the accompanying poster, we briefly outline the core pathway and its regulation, and describe the breadth of its roles in animal development.

## Introduction

The Hippo pathway was identified using *Drosophila* genetic mosaic screens for genes that regulate cell number (Kim and Jho, 2018). Subsequent studies in vertebrates highlighted the conservation of the Hippo pathway and its involvement in tissue growth, regeneration and tumorigenesis (Moya and Halder, 2018). The core pathway consists of a kinase cascade in which the upstream Ste20 family kinase Hippo (Hpo; MST1/2 in mammals), together with its binding partner Salvador (Sav; Sav1 in mammals), phosphorylates and activates the NDR family kinase Warts (Wts; LATS1/2 in mammals) and its scaffold protein Mob1 as tumour suppressor (Mats; MOB1A/B in mammals) (Kim and Jho, 2018; Misra and Irvine, 2018). Wts/LATS in turn phosphorylates and inhibits the transcriptional co-activator Yorkie (Yki, YAP/TAZ in mammals). Phosphorylation by Wts/LATS restricts Yki or YAP/TAZ nuclear accumulation by promoting its interaction with 14-3-3 proteins, thereby sequestering Yki/YAP/TAZ in the cytoplasm, as well as decreasing nuclear import and/or increasing nuclear export (Misra and Irvine, 2018). In the nucleus, Yki/YAP/TAZ interacts with the TEA domain transcription factor Scalloped (Sd; TEAD1-4 in mammals), promoting the expression of cell growth and proliferation (e.g. DNA replication, mitosis and chromosome organisation), stem cell identity and tissue architecture (e.g. cytoskeleton, extracellular matrix) genes (Hansen et al., 2015). Although the pathway is often referred to as the ‘Hippo’ pathway, it is important to stress that Yki/YAP/TAZ can be regulated independently of the core kinase cascade by numerous upstream inputs (Piccolo et al., 2014). This includes other kinases that phosphorylate Yki/YAP/TAZ directly [e.g. 5′ AMP-activated protein kinase (AMPK), Abelson tyrosine-protein kinase (Abl) and proto-oncogene tyrosine-protein kinase Src], as well as the actin cytoskeleton (Piccolo et al., 2014). In many cases, it is not clear whether developmental changes in Yki/YAP/TAZ activity are due to alterations in core kinase cascade activity, activity of other kinases or changes in the cellular microenvironment.

**Figure:**
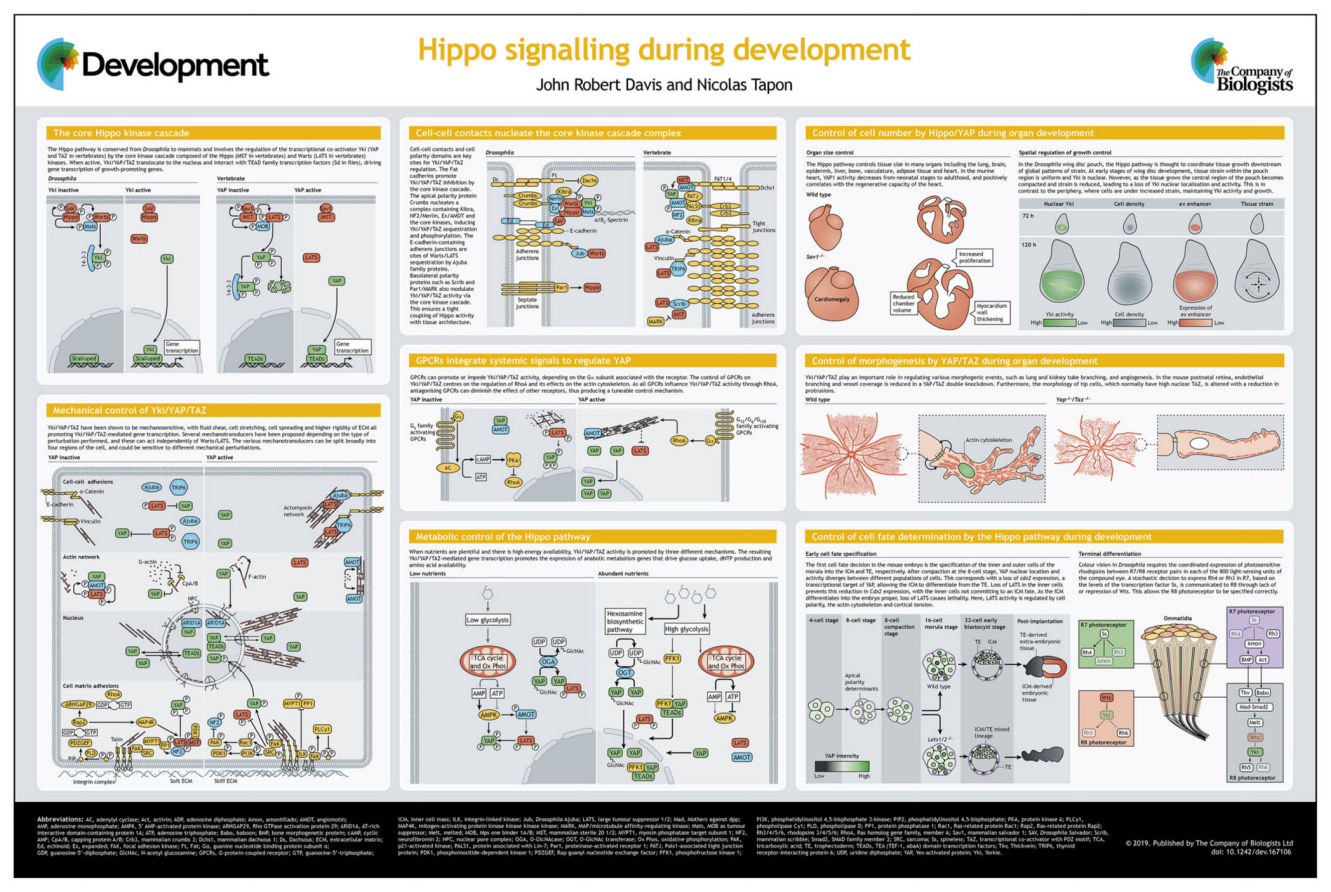


Mutations in genes that repress Yki/YAP/TAZ activity lead to extensive tissue over-growth (Moya and Halder, 2018), highlighting their potent growth-promoting function. It is therefore not surprising that Yki/YAP/TAZ activity is tightly regulated by known upstream inputs, including the cell’s nutrient/energy status, the physical environment, tissue architecture (cell-cell and cell-ECM contacts, cell polarity) and extrinsic biochemical signals (Fulford et al., 2018). This suggests that Yki/YAP/TAZ represents a nexus of cell signalling, integrating a range of local and systemic information to control a host of cell behaviours. Although initially identified as a growth regulator, it has become evident that the Hippo pathway also regulates other developmental processes, such as morphogenesis and cell-fate determination. Here, and in the accompanying poster, we provide an overview of the regulation of Yki/YAP/TAZ and the Hippo pathway, and highlight examples of their roles in vertebrate and *Drosophila* developmental processes.

## Regulation of Yki/YAP/TAZ by cell polarity

In epithelia, the Hippo pathway is proposed to act as a sensor of tissue organisation (Karaman and Halder, 2018). Indeed, the regulation of Yki/YAP/TAZ is intimately coupled to hallmarks of tissue architecture such as cell polarity, and cell-cell and cell-extracellular matrix (ECM) contacts (Genevet and Tapon, 2011). Several key components of the pathway form a complex localised in the sub-apical region (SAR) in flies, and in tight junctions (TJs) in vertebrates. These include the FERM domain protein Merlin/neurofibromin 2 (NF2), the scaffold protein Kidney and brain protein (Kibra) and the actin-associated protein angiomotin (Amot, or its proposed fly functional homologue Expanded – Ex), which recruit the core kinases Hpo/MST and Wts/LATS (Fulford et al., 2018). The SAR/TJs therefore act as hubs where the core kinases assemble into an active complex that promotes Yki/YAP/TAZ inhibitory phosphorylation. In addition, Amot and Ex directly bind Yki/YAP/TAZ and are believed to sequester it away from the nucleus (Badouel et al., 2009; Chan et al., 2011; Oh et al., 2009; Oka et al., 2012; Varelas et al., 2010; Zhao et al., 2011). As the SAR and TJs are key regulatory sites for apico-basal polarity, interfering with apical polarity determinants often leads to Yki/YAP/TAZ activation by disrupting the localisation and activity of Hpo/MST and Wts/LATS (Schroeder and Halder, 2012).

Basolateral polarity factors also modulate Yki/YAP/TAZ activity, particularly Scribble, which scaffolds and activates the core kinase cascade in mammals, and Par-1/MAP/microtubule affinity-regulating kinases 1-4 (MARK1-4), which represses core kinase activity in both flies and mice (Cordenonsi et al., 2011; Heidary Arash et al., 2017; Huang et al., 2013; Kwan et al., 2016; Mohseni et al., 2014). Polarity in epithelial cells requires the formation of stable cell-cell contacts, such as the E-cadherin-containing adherens junctions (AJs). Several AJ components, such as α-catenin and Ajuba-Zyxin family proteins, have been proposed to regulate Yki/YAP/TAZ dynamics (Fulford et al., 2018). Finally, the Fat atypical cadherins, which are implicated in planar cell polarity, repress Yki/YAP/TAZ activity via the core kinase cascade (Blair and McNeill, 2018). Thus, Yki/YAP/TAZ activity is tightly coupled to tissue architecture, presumably to allow homeostatic control of tissue repair in response to injury.

## Mechanical control of Yki/YAP/TAZ

Yki/YAP/TAZ are mechanosensitive, becoming transcriptionally active when cells are exposed to rigid ECM (Dupont et al., 2011), are spread over a large substrate surface area (Dupont et al., 2011; Nardone et al., 2017; Wada et al., 2011), are mechanically stretched (Aragona et al., 2013) or experience fluid shear (Nakajima et al., 2017). Interestingly, genes important for ECM composition, actin dynamics and adhesion formation have all been identified as being Yki/YAP/TAZ target genes (Calvo et al., 2013; Nardone et al., 2017). This could function as a feedback mechanism to regulate the sensitivity of Yki/YAP/TAZ to mechanical stimuli, or as a cellular adaptation to mechanical strain. Several mechanotransducers regulating Yki/YAP/TAZ activity have been identified, some independent of and others dependent on MST or LATS (Meng et al., 2016; Panciera et al., 2017). Additionally, other Ste20-family kinases, such as Happyhour in *Drosophila* and mitogen-activated protein kinase kinase kinase kinases 1, 2, 3 and 5 (MAP4K1, MAP4K2, MAP4K3 and MAP4K5) in mammals have also been shown to redundantly regulate Yki/YAP/TAZ dynamics downstream of actin cytoskeletal dynamics (Meng et al., 2015; Zheng et al., 2015). The mechanotransducers identified so far can be broadly split into four categories based on their location within the cell: cell-cell adhesions, cytoplasmic actin cytoskeleton, nucleus and cell-ECM adhesions.

## Cell-cell adhesions

High cell density activates the core kinase cascade (Zhao et al., 2007) via the AJs (Kim et al., 2011). In *Drosophila* and mammalian systems, vinculin (Dutta et al., 2017) and α-catenin (Ibar et al., 2018; Rauskolb et al., 2014) relay mechanical information from cell-cell contacts to the cytoplasm through conformational changes. When placed under tension, these proteins unfold, releasing hidden binding sites. Vinculin unfolding exposes a binding site for thyroid receptor interacting protein 6 (TRIP6) (Dutta et al., 2017), and α-catenin unfolding reveals binding sites for Ajuba in *Drosophila* or the Ajuba family member LIM domain containing protein 1 (LIMD1) in mammals (Ibar et al., 2018; Rauskolb et al., 2014). Both TRIP6 and Ajuba/LIMD1 sequester Wts/LATS to AJs, and inactivate its kinase activity (Dutta et al., 2017; Ibar et al., 2018; Rauskolb et al., 2014). Interestingly, the disruption of either TRIP6 or LIMD1 leads to loss of LATS localisation to AJs and nuclear YAP (Dutta et al., 2017; Ibar et al., 2018). This raises questions about whether TRIP6/Ajuba/LIMD1 act independently or synergistically, and whether they are crucial for AJ dynamics upon mechanical strain.

## Cytoplasmic actin cytoskeleton

The actomyosin cytoskeleton is the major mechanical component of cells, and is required for cells to respond to external forces and to generate internal forces to resist strain. The role of the actomyosin cytoskeleton for Yki/YAP/TAZ dynamics has been highlighted in numerous studies; however, the exact mechanism has not been elucidated. Several studies support actin polymerisation or F-actin stability as factors that promote Yki/YAP/TAZ activity (Gaspar and Tapon, 2014). Loss of proteins known to antagonise actin polymerisation, such as capping proteins (Aragona et al., 2013; Fernandez et al., 2011; Sansores-Garcia et al., 2011) and Cofilin/Twinstar (Ko et al., 2016; Sansores-Garcia et al., 2011), or expression of constitutively active actin polymerisation proteins, such as Diaphanous (Sansores-Garcia et al., 2011), all increase Yki/YAP/TAZ activity. Conversely, treatment with Latrunculin A, a drug that prevents actin polymerisation, reduces Yki/YAP/TAZ activity (Aragona et al., 2013; Das et al., 2016; Dupont et al., 2011). However, it is unclear whether these changes in Yki/YAP/TAZ activity are due to actin polymerisation or to other factors, such as actin network stability. Thus, although there is clear evidence that an increase in F-actin promotes Yki/YAP/TAZ activity, the molecules that mediate this are currently unknown. In mammals, the Amot-family proteins Amot-p130, AmotL1 and AmotL2 (hereafter just Amot) have been identified as a link between actin and YAP regulation. Amot can directly bind to YAP/TAZ and sequester it to TJs, independently of its phosphorylation by LATS1/2 (Zhao et al., 2011). Amot also binds to F-actin, which competes with YAP for Amot binding (Chan et al., 2013; Dai et al., 2013; Mana-Capelli et al., 2014). Therefore, F-actin accumulation can displace Amot from YAP and promote YAP nuclear entry. Interestingly, Amot is phosphorylated by LATS, which promotes its association with YAP by displacing Amot from F-actin (Chan et al., 2013; Mana-Capelli et al., 2014). The Amot-YAP inhibitory interaction can be disrupted by shear stress in zebrafish blood vessels (Nakajima et al., 2017), but whether Amot acts as a YAP regulator for other types of physiological mechanical cues remains to be tested.

## Nucleus

The activity of Yki/YAP/TAZ is dependent on balancing the regulation of their nuclear import and export rates by various factors located either in the cytoplasm or nucleus (Cho et al., 2018; Ege et al., 2018; Manning et al., 2018). YAP nuclear import rates can be modified by nuclear deformation (Elosegui-Artola et al., 2017). For example, deforming the nucleus using an AFM probe can induce YAP nuclear localisation, even when cells are treated with Latrunculin A (Elosegui-Artola et al., 2017). Likewise, loss of nesprins [components of the linker of nucleoskeleton and cytoskeleton (LINC) complex that connect the nucleus to the actin cytoskeleton] blocks YAP nuclear localisation when cells are cultured on stiff substrates (Elosegui-Artola et al., 2017). This mechanism of increasing nuclear import rates is not specific to YAP, however, and is a result of stretched nuclear pore complexes reducing their steric hindrance on active nuclear import (Elosegui-Artola et al., 2017). Interestingly, the mechanosensitive kinase Src has been shown to reduce YAP nuclear export rates by inhibiting the export protein exportin-1 (XPO1), adding another layer of regulation of YAP nuclear dynamics after mechanical stimuli (Ege et al., 2018). In *Drosophila*, Yki nuclear import rates are suppressed by Wts (Manning et al., 2018). Nuclear YAP is also regulated upon mechanical manipulations via the SWI/SNF-like BRG1/BRM-associated factor (BAF) nucleosome remodelling complex. YAP interacts with the BAF complex member AT-rich interactive domain-containing protein 1A (ARID1A), and this association prevents YAP binding to TEADs (Chang et al., 2018). The BAF complex can also bind to nuclear F-actin via one of the other complex components, Brahma-related gene 1 (BRG1) (Rando et al., 2002). When cells are cultured on rigid substrates, F-actin accumulates in the nucleus, reducing YAP sequestration by ARID1A and allowing its association with TEADs (Chang et al., 2018). However, the mechanism for competitive binding between F-actin and YAP to the BAF complex is unknown.

## Cell-ECM adhesions

In mammalian systems, the composition and rigidity of the ECM is an important regulator of YAP/TAZ activity (Dupont et al., 2011). When cells are cultured on soft substrates (<3 kPa) YAP/TAZ are cytoplasmic; however, their nuclear localisation increases when cultured on rigid substrates (>10 kPa) (Dupont et al., 2011; Elosegui-Artola et al., 2016). This dependence on matrix rigidity can be mitigated by matrix composition, with cells cultured on Agrin-containing soft matrices exhibiting high YAP nuclear localisation (Chakraborty et al., 2017). This is presumably mediated through matrix-specific integrin heterodimers seeding focal adhesion complexes with variable mechanical and biochemical properties (Seetharaman and Etienne-Manneville, 2018). Indeed, fibronectin and laminin matrices on glass coverslips elicited different YAP responses (Kim and Gumbiner, 2015). Recently, integrin clustering was also shown to modulate how YAP responds to matrix rigidity (Oria et al., 2017), further highlighting the complex nature of cell-ECM dynamics. Cell spreading has also been implicated in regulating YAP/TAZ (Dupont et al., 2011), although it is unclear whether this occurs through altered actin dynamics, nuclear deformation or integrin signalling per se (Brusatin et al., 2018; Nardone et al., 2017).

How matrix rigidity can be transformed into biochemical signals has been examined in various biological settings, and a general model of tension-mediated conformational protein changes and adhesion maturation has emerged (Klapholz and Brown, 2017). Indeed, Talin, the protein connecting integrins to F-actin, has been reported to act as a molecular tension sensor, linking actomyosin contraction to matrix rigidity, which promotes YAP/TAZ activity on rigid substrates (Elosegui-Artola et al., 2016). The exact molecular mechanism by which Talin conformational changes lead to YAP activation is currently unknown, but they could promote focal adhesion signalling. Supporting this hypothesis is the myriad of focal adhesion signalling molecules, such as Focal Adhesion Kinase (FAK) (Kim and Gumbiner, 2015), Integrin-Linked Kinase (ILK) (Serrano et al., 2013), the PDZ and LIM Domain Containing proteins PDLIM5 (Enigma-like)/PDLIM7 (Enigma) (Elbediwy et al., 2018), and Src-family tyrosine kinases (Kim and Gumbiner, 2015), which have all been linked to YAP regulation. Most of these molecules promote YAP activity; for example, Src has been suggested to both directly [via phosphorylation of the C-terminal region of YAP (Li et al., 2016)], and indirectly [by inhibiting Nf2 or LATS (Fan et al., 2013; Kim and Gumbiner, 2015; Si et al., 2017)] promote YAP nuclear localisation. Recently, the Ras-related GTPase Rap2 was reported to reduce YAP nuclear accumulation when cells are grown on soft substrates (Meng et al., 2018). In the absence of FAK activity on soft substrates, phosphatidlyinositol-4,5,biphosphate (PIP_2_) levels increase, leading to Rap2 activating MAP4K4/6/7 to phosphorylate and activate LATS, independently of MST1/2 (Meng et al., 2018). The increasing list of focal adhesion components that have been linked to YAP regulation could suggest high levels of redundancy to maintain tight control of YAP activity or could highlight the sophisticated signalling machinery that allows focal adhesions to respond appropriately to a wide variety of substrate compositions and mechanics.

## GPCRs and the YAP/TAZ regulation

Yki/YAP/TAZ activity can also be controlled by extrinsic biochemical signals such as G-protein-coupled receptors (GPCRs), which interact with cytoplasmic heterotrimeric G proteins composed of α, β and γ subunits. YAP/TAZ function downstream of GPCR signalling in mammalian cells both in culture (Miller et al., 2012; Yu et al., 2012) and *in vivo* (Serafimidis et al., 2017). In culture, the presence of lysophospholipids sphingosine 1-phophate (S1P) and lysophosphatidic acid (LPA) in serum induces an increase in nuclear localisation and transcriptional activity of YAP (Miller et al., 2012; Yu et al., 2012). This is mediated through activation of their respective GPCRs, S1P_2_ and LPA1/3, both of which interact with the G_12_ family of Gα subunits (Miller et al., 2012; Yu et al., 2012). Other Gα subfamilies (G_q_ and G_i/0_) also promote YAP transcriptional activity (Yu et al., 2012, 2013). Indeed, activating mutations in G_q_ cause YAP-dependent uveal melanoma (Feng et al., 2014; Yu et al., 2014). Interestingly, GPCR signalling through the G_s_ family inhibits Yki/YAP/TAZ activity (Yu et al., 2012). The effect of GPCRs on YAP is mediated through Rho small GTPases, with the different Gα-family proteins either promoting or repressing RhoA activity (Yu et al., 2012, 2013). Mechanistically, the G_12_ and G_q_ families regulate RhoA by upregulating RhoGEF/Trio activity, which in turn promotes the GTP-bound kinase active form of RhoA/Rac (Feng et al., 2014; Yu et al., 2012). G_s_ family proteins inhibit RhoA activity through activation of adenylyl cyclases, which increases cAMP levels and in turn activates PKA, a known inhibitor of RhoA (Yu et al., 2012, 2013). The effect of RhoA on YAP activity is dependent on the actomyosin network, but exactly how is still under debate. Both actomyosin-dependent repression of LATS and LATS-independent displacement of YAP from Amot by F-actin have been proposed (Feng et al., 2014; Miller et al., 2012; Yu et al., 2012). The convergence of different GPCR signals on RhoA activity provides a mechanism for extrinsic signals to mitigate the effect of one another. Indeed, LPA (which activates G_12_) and epinephrine (which activates G_s_) cancel the ability of one another to regulate YAP activity (Yu et al., 2012). Therefore, the effects of GPCR signalling on YAP can be regulated through altering the levels of activation of different GPCR sub-groups.

## Cell metabolism and the Hippo pathway

As Yki/YAP/TAZ activity is known to regulate growth, it is not surprising that this activity is regulated by energy-sensing and nutrient availability pathways. YAP is a phospho-target of AMPK, a known energy sensor that is sensitive to AMP levels (Deran et al., 2014; Gailite et al., 2015; Mo et al., 2015; Wang et al., 2015). AMPK directly inhibits YAP activity, but it also stabilises AmotL1, which promotes the activity of LATS, further reducing YAP transcription (Deran et al., 2014). Additionally, the glycolytic enzyme phosphofructokinase (PFK1) promotes YAP-mediated gene transcription by binding to TEADs (Enzo et al., 2015). When glycolysis is reduced, PFK1 levels decrease and are unable to promote YAP transcription. The nutrient-sensitive hexosamine biosynthetic pathway (HBP) has also been shown to regulate YAP (Peng et al., 2017; Zhang et al., 2017). HBP converts glucose, glucosamine and acetyl-CoA into uridine diphosphate N-acetyl glucosamine (UDP-GlcNAc). YAP is O-GlcNAcylated by O-GlcNAcylation transferase (OGT), which requires UDP-GlcNAc as a donor (Peng et al., 2017; Zhang et al., 2017). O-GlcNAcylation of YAP prevents LATS binding, therefore promoting YAP activity. When nutrients are scarce, O-GlcNAcylation of YAP can be reversed by O-GlcNAcase (OGA) to release the O-GlcNAcyl group, reinstating LATS-mediated inhibition of YAP (Peng et al., 2017). Other metabolic pathways regulate YAP activity via upstream effectors or by modulating YAP/TAZ protein levels. For example, RhoA requires modification by the mevalonate pathway to be active (Sorrentino et al., 2014) and TSC downregulation leads to an increase in YAP/TAZ protein levels through a reduction in autophagy (Liang et al., 2014). Interestingly, YAP/TAZ transcription targets include genes important for anabolic metabolism, specifically glucose uptake (Cox et al., 2018), dNTP production (Santinon et al., 2018) and amino acid availability (Bertero et al., 2018). This highlights the importance of Yki/YAP/TAZ in linking energy and nutrient levels with the anabolic requirements of growth.

## The role of Hippo/YAP signalling in growth control

Hippo signalling was first described as a growth control pathway and has since been linked to the regulation of tissue size in a plethora of model organisms. In vertebrates, YAP/TAZ activity has been linked to cell number control in the developing lung (Lin et al., 2015), brain (Lavado et al., 2018), epidermis (Lee et al., 2008; Schlegelmilch et al., 2011), liver (Song et al., 2010), bone (Goto et al., 2018), vasculature (Astone et al., 2018), adipose tissue (An et al., 2013) and heart (Heallen et al., 2011). Indeed, YAP/TAZ expression is often high in stem/progenitor compartments in many vertebrate tissues, such as the liver, skin, gut and heart (Mo et al., 2014). In the mouse liver, YAP overexpression (Camargo et al., 2007; Dong et al., 2007) or disruption of the core kinase cascade (Chen et al., 2015; Zhang et al., 2010; Zhou et al., 2009) promotes increased adult organ size, but it is not clear what role YAP/TAZ play during normal liver developmental growth. In *Drosophila*, Hippo/Yki activity regulates cell number in the imaginal discs (the larval precursors of adult appendages), as well as the CNS (Gailite et al., 2015; Poon et al., 2016; Reddy and Irvine, 2011; Reddy et al., 2010; Weynans et al., 2016). In some of these tissues, such as the murine heart, YAP activity and protein levels change throughout development, and this correlates with the proliferative capacity of the tissue (Morikawa et al., 2017; von Gise et al., 2012). Conditional knockdown of YAP1 in embryonic hearts leads to a reduction in cardiomyocyte number, highlighting the importance of YAP1 in heart developmental growth (von Gise et al., 2012). Interestingly, YAP expression decreases from neonates, when the heart has fully developed, throughout adulthood (von Gise et al., 2012), and this is linked with an increase in YAP inhibitory phosphorylation (Heallen et al., 2013). The change in YAP activity is due in part to changes in ECM composition. The heparan sulfate proteoglycan agrin shows reduced levels around the heart between P1 and P7 in mouse neonates (Bassat et al., 2017). Agrin binds to dystroglycan (DAG1), a member of the inhibitory dystrophin-glycoprotein complex (DGC) that normally sequesters YAP (Morikawa et al., 2017); this association promotes YAP dissociation from the DGC (Bassat et al., 2017). Decreasing YAP protein level and activity are linked to a reduction in the regenerative capacity of the tissue, with adult murine hearts being unable to repair after myocardial infarction (Xin et al., 2013). Increasing YAP activity in adult hearts restores the neonatal and juvenile regenerative capacity of the heart (Xin et al., 2013), further highlighting the importance of the Hippo pathway in growth control.

While the Hippo pathway regulates organ size in many tissues, the upstream signals involved in its physiological regulation in these different contexts often remain elusive. An exception is the *Drosophila* wing imaginal disc where several studies have assessed the spatial and temporal control of Yki activity and tissue growth. In early larval stages, Yki is nuclear and active across the entire wing disc pouch (Pan et al., 2018). However, as larvae develop, Yki activity (as reported by expression of the Yki/Sd target gene *expanded*) decreases in the centre of the wing pouch but is maintained at the periphery (Pan et al., 2018). This mirrors Yki localisation, with an overall reduction of nuclear Yki in the centre of the wing pouch, except at the DV boundary (Pan et al., 2018). This change in activity correlates with the spatial variation in tissue tension at the latter stages of larval wing disc development, with the periphery experiencing greater levels of anisotropic tension (Legoff et al., 2013; Mao et al., 2013). Furthermore, mechanical stretching of *ex vivo* cultured wing discs showed an increase in cell proliferation (Schluck et al., 2013). These observations have led to the idea that tension-mediated control of the Hippo pathway coordinates cell proliferation across the wing disc (Fletcher et al., 2015; Pan et al., 2016, 2018). Specifically cells in the centre of the wing pouch experience reduced tension as development progresses, leading to reduced Yki activity and proliferation, whereas cells at the periphery experience greater tension and continue to proliferate (Fletcher et al., 2015; Pan et al., 2016). α-catenin-Ajuba (Alégot et al., 2019) and β-spectrins-Hpo (Fletcher et al., 2015) have been proposed as mediators of the effects of tissue tension on Hippo pathway activity. Recent findings have challenged this model, suggesting that Notch represses *ex* expression in the centre of the wing pouch, while Yki/Sd drives its expression uniformly throughout the disc rather than in a tension-dependent pattern (Djiane et al., 2014; Wang and Baker, 2018).

## The role of Yki/YAP/TAZ in regulating morphogenesis

Given the ability of Yki/YAP/TAZ to modulate the cytoskeleton and ECM composition, it is perhaps unsurprising that Hippo signalling is increasingly being linked to tissue morphogenesis. In vertebrate systems, YAP/TAZ have been shown to promote branching in the lung (Lin et al., 2017) and vasculature (Astone et al., 2018), but can also inhibit branching in the kidney (Reginensi et al., 2016). The Hippo pathway has also been implicated in regulating cell rearrangements during mouse retinal angiogenesis (Neto et al., 2018), *Drosophila* tracheal morphogenesis (Ghabrial et al., 2011; Poon et al., 2018) and *Drosophila* border cell migration, in this case via phosphorylation of the actin polymerisation factor Enabled rather than Yki (Lucas et al., 2013). In most of these systems, Yki/YAP/TAZ is believed to increase cell migration by upregulating actin cytoskeleton and ECM genes (Wang et al., 2017). In postnatal mouse retinal angiogenesis, double knockdown of YAP and TAZ severely reduces vessel coverage and sprouting (Neto et al., 2018), and this is accompanied by a morphological change in tip cells, which show drastically reduced protrusions and branching (Sakabe et al., 2017). Likewise, silencing of YAP/TAZ in cultured endothelial cells reduces their migration, even when stimulated with the promigration factor VEGF (Neto et al., 2018; Sakabe et al., 2017; Wang et al., 2017), further highlighting the importance of YAP/TAZ for migration. Interestingly, there is a possible divergence in the role of YAP/TAZ during angiogenesis: TAZ is nuclear in tip cells, whereas YAP is nuclear in the remodelling vessels (Neto et al., 2018). It would be interesting to examine whether the two paralogues have differing gene transcription profiles or upstream regulatory inputs that could explain why there is divergence in activity.

## The role of Yki/YAP/TAZ in regulating cell fate

Yki/YAP/TAZ have been implicated in an increasing number of cell fate decisions. In mammals, YAP and TAZ have been linked to cell specification in the lungs (Mahoney et al., 2014), mammary glands (Chen et al., 2014; Skibinski et al., 2014), pancreas (Gao et al., 2013; Mamidi et al., 2018), kidneys (McNeill and Reginensi, 2017) and liver (Zhang et al., 2010). In most systems, a reduction in YAP/TAZ activity is required for correct differentiation, such as in the mammary glands (Chen et al., 2014), pancreas (Gao et al., 2013) and kidney (McNeill and Reginensi, 2017), with YAP/TAZ maintaining pluripotent characteristics (Lian et al., 2010; Taz et al., 2016). However, in some specific cases, YAP activity is required for correct specification, such as the ductal cells in the mouse liver (Zhang et al., 2010) or the pre-implantation mouse embryo. The first cell-fate decision in mouse development is between the trophectoderm (TE, which gives rise to extra-embryonic tissues) and the inner cell mass (ICM, which gives rise to the embryo proper). This decision is driven through the regulation of caudal-type homeobox protein 2 (Cdx2), a TE cell fate determinant that is a downstream transcriptional target of YAP/TEADs (Nishioka et al., 2009). At the four-cell stage, YAP distribution is both cytoplasmic and nuclear in all cells. Cells then undergo asymmetric divisions that produce polarised and unpolarised cells, which will specify the TE or ICM lineages, respectively (Anani et al., 2014). The apical domain in polarised TE cells promotes YAP activity by sequestering Amot away from the basolateral domain, where it promotes activity of the core kinase cascade along with Nf2 (Cockburn et al., 2013; Hirate et al., 2015; Shi et al., 2017) and binds to YAP to limit nuclear import (Leung and Zernicka-Goetz, 2013). Polar cells also have higher cortical tension (Maître et al., 2016), and the change in Amot localisation could be mediated through an increase in F-actin at the apical domain (Anani et al., 2014; Leung and Zernicka-Goetz, 2013). Intriguingly, differential cortical tension is important for cell positioning, with polar cells surrounding apolar cells (Anani et al., 2014; Maître et al., 2016). As YAP activity is known to promote actomyosin contraction (Calvo et al., 2013), it is possible that higher YAP activity also aids cell positioning in the blastocyst. Ultimately, YAP activity in outer polar cells maintains Cdx2 expression, while loss of YAP activity in the apolar inner cells leads to Cdx2 downregulation (Nishioka et al., 2009). Accordingly, LATS1/2 knockdown prevents repression of YAP activity and Cdx2 expression in inner cells, causing them to acquire a TE-like fate (Lorthongpanich et al., 2013; Nishioka et al., 2009).

In the *Drosophila* retina, Yki has been implicated in terminal differentiation of the R8 photoreceptor cell type, which is required for colour vision (Rister et al., 2013). During retinal development, R8 cells receive a signal from neighbouring R7 cells, 35% of which stochastically secrete active BMP and Activin ligands (Thanawala et al., 2013; Wernet et al., 2015). These ligands in turn signal to the R8 to tip the balance of a bistable negative-feedback loop between Wts and the insulin signalling modulator Melted (Melt) (Mikeladze-Dvali et al., 2005). If the R8 receives the BMP/Activin signal, Melt shuts down Wts expression, Yki is activated and switches on the expression of Rhodopsin 5. In the absence of BMP/Activin ligands, Wts prevails, Yki is repressed and Rhodospin 6 is expressed instead (Jukam et al., 2013; Mikeladze-Dvali et al., 2005; Wells et al., 2017). Thus, directional signalling between the R7 and R8 cells mediated by crosstalk between the BMP/Activin and Hippo pathways leads to a stable cell fate choice.

## Concluding remarks

Although it was first discovered through its role in tissue growth, the Hippo pathway and its transcriptional effectors Yki/YAP/TAZ have emerged as key regulators of numerous developmental decision-making processes, including altering cell behaviour during tissue morphogenesis, as well as cell fate. This functional diversity implies context-specific regulatory inputs, as well as transcriptional outputs. Cell-specific upstream signalling is likely achieved through cellular context (as in the cell polarity-dependent TE/ICM fate choice in early mouse embryos), crosstalk with other pathways (as for *Drosophila* photoreceptor specification), as well as tissue-specific upstream signalling. Likewise, the field will need to unravel how the Yki/YAP/TAZ transcriptional programme is refined tissue specifically and how it intersects with that of other developmental signals. This is a particular challenge for vertebrate systems, where the relative contributions of the two co-activators YAP and TAZ (with a number of cell-specific alternative transcripts) and the four TEAD family members remains to be defined in many developmental contexts. However, with an improving understanding of the transcriptional machinery mobilised by Yki/YAP/TAZ and the increasing use of *in vivo* genomic engineering, we can look forward to progress in this fascinating field.
